# Bridging the gap in flat optics: the dawn of partially nonlocal metasurfaces

**DOI:** 10.1038/s41377-026-02397-0

**Published:** 2026-07-14

**Authors:** Jingxuan Wei, Yong Liu

**Affiliations:** School of Optoelectronic Science and Engineering, University of Electronic Science and Technology of China, Chengdu, 611731 China

**Keywords:** Nanophotonics and plasmonics, Sub-wavelength optics

## Abstract

Overcoming the traditional trade-off between spatial control and high quality factors, a new class of multifunctional photonic crystals bridges the gap between local and nonlocal light manipulation. This work opens a new frontier in flat optics: the realm of partially nonlocal metasurfaces, paving the way for advanced imaging, communication, and analog optical computing.

Flat optics has reshaped micro- and nano-photonics by enabling subwavelength control of phase, amplitude, and polarization on a planar platform^1–3^. Yet the field has long been trapped between two extreme paradigms. Traditional metasurfaces rely on *local* modulation through spatially varying subwavelength structures^4,5^, granting enormous spatial design freedom but suffering from excessive radiative leakage and consequently low quality factors (Q factors). In contrast, photonic crystal (PhC) structures supporting *nonlocal* modes, most notably bound states in the continuum (BICs), achieve ultra-high Q factors through minimal leakage channels^6–9^, but their response is governed by global periodicity, severely limiting the capacity for complex spatial light field encoding. A central challenge has therefore been to accommodate both high Q factors and high spatial modulation degrees of freedom (DoFs) on a single platform.

This challenge also conceals a deeper physical question: is it possible to encode spatially varying information into a nonlocal structure without fundamentally degrading its collective resonance? Or does any spatial modulation inevitably compromise nonlocality? In a study recently published in *Light: Science & Applications*^10^, a joint team from Tsinghua University and the National University of Singapore provides a compelling practical answer to this question.

The researchers proposed and experimentally realized “Local-nonlocal assisted multifunctional photonic crystals” in a single-layer titanium dioxide (TiO_2_) device, as shown in Fig. 1. By embedding locally tunable meta-notches within photonic crystal pillars, they achieved efficient 2π local phase coverage utilizing topological phases assisted by spectral singularities^5^. The key physical insight is that the BIC mode exhibits a field minimum precisely at the notch location, rendering the nonlocal resonance largely immune to the local structural perturbation. Experimentally, they demonstrated wavelength-selective, high-contrast holographic imaging (genus 1 and genus 2 patterns) as evidence of local phase control, and confirmed via angle-resolved band structure measurements that the device preserves a near-perfect BIC even when strict periodicity is broken. This achievement realizes the integration of arbitrary wavefront shaping and high-Q resonances on a single platform.

**Fig. 1:**
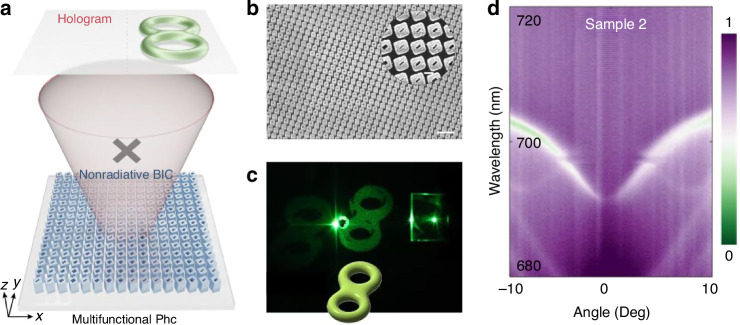
. **Experimental realization of local-nonlocal assisted multifunctional photonic crystals**. **a** Conceptual schematic of the unified planar platform, demonstrating simultaneous support for nonlocal bound states in the continuum (BICs) and local wavefront shaping. **b** Scanning electron microscope (SEM) image of the fabricated TiO_2_ nanopillar array with spatially varying embedded meta-notches. **c** Real-space control: Experimental optical photograph of the projected high-contrast meta-hologram (a genus 2 image), highlighting the device’s capability for arbitrary local wavefront shaping in the spatial domain. **d** *k*-space control: Measured angle-resolved band structure confirming the robust preservation of the unperturbed nonlocal BIC resonance at the Γ point, demonstrating stable modulation in momentum space despite the broken strict periodicity

While this work achieves remarkable device performance, it raises a more fundamental physical question: where does this design DoF actually come from? Is it a perfect superposition of two independent mechanisms, or does it reflect a deeper physical trade-off?

To address this question, we introduce an information-theoretic perspective. In flat optics, modulation DoFs are intimately connected to the spatial correlation between structural units (see Fig. 2a). For local metasurfaces, each pixel can be designed independently with negligible inter-unit correlation, permitting a large total information encoding capacity. At the other extreme, the response of a perfectly nonlocal BIC is global, with the light field spatially correlated across the entire device, shifting the modulation entirely to momentum (*k*) space^2^. The transition from a non-periodic structure to a strictly periodic one, accompanied by a drastic reduction in independent design parameters, inevitably leads to a precipitous drop in spatial encoding capacity.

**Fig. 2:**
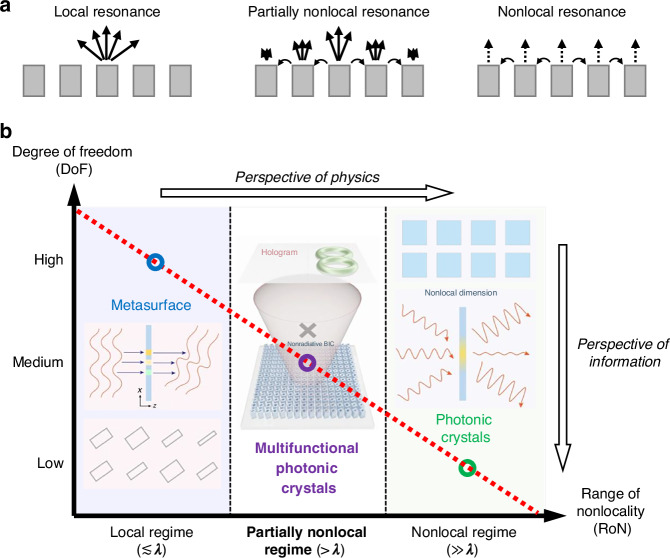
. **Physical and informational perspectives of flat optics**. **a** Schematics of local, partially nonlocal, and nonlocal resonances, illustrating the varying degrees of spatial correlation between structural units. **b** A schematic two-dimensional map relating the Degree of Freedom (DoF, defined as total information encoding capacity) to the Range of Nonlocality (RoN, defined as the spatial coherence length of the dielectric profile). The partially nonlocal regime (multifunctional photonic crystals) occupies the intermediate territory between traditional local metasurfaces and nonlocal photonic crystals. The positions of each paradigm are illustrative and intended to convey the qualitative trend

To map these physical and informational perspectives, we assign the “Range of Nonlocality” (RoN), which is defined here as the spatial coherence length of the dielectric profile in the presence of inter-unit coupling, to the *x*-axis, and the “Degree of Freedom” (DoF), that represents the total information encoding capacity of the device, to the *y*-axis, as shown in Fig. 2b. Two poles of flat optics then become apparent:

1. **Local regime:** RoN is comparable to or smaller than the wavelength, with negligible coupling between units, and the DoF is extremely high.
2. **Nonlocal regime:** RoN far exceeds the wavelength, the system is dominated by global periodicity, and the DoF is extremely low.

A natural question then arises: what occupies the vast, largely unexplored territory between these two extremes? This is precisely the frontier opened by this work^10^: *partially nonlocal metasurfaces*. While the authors describe their device through the functionally oriented concept of “multifunctional photonic crystals”, their design fundamentally embodies this novel physical paradigm.

A crucial subtlety deserves emphasis here. A perfect PhC BIC, with a truly infinite Q factor, is a mathematical construct defined for an idealized, infinitely periodic structure. Any real device, by virtue of its finite size and inevitable imperfections, already operates as a quasi-BIC (qBIC) with a finite (though potentially very large) Q-factor. In this sense, any experimental photonic crystal already inhabits the partially nonlocal regime. The key insight is that in this regime, strict spatial periodicity is not fully exploited: it represents, in information-theoretic terms, a redundant use of the available design space. The meta-notch strategy of Lv et al. capitalizes on precisely this redundancy. By introducing controlled local variations that fall within the perturbation tolerance of the qBIC mode, specifically at positions where the modal field is minimal, the authors reclaim spatial DoFs that were previously “wasted” by unnecessary strict periodicity, without measurably degrading the collective resonance.

This perspective also clarifies why the experimentally measured band structure still exhibits vanishing linewidth despite the broken periodicity. The spatially modulated device supports what is effectively a qBIC whose lateral coherence length, while no longer formally infinite, remains much larger than the wavelength. It is this residual nonlocality (rather than perfect periodicity) that sustains the strong angular dispersion observed in the measurements. The qBIC retains its pronounced angle-dependent character because the inter-unit coupling still extends over many lattice periods, ensuring that the mode “samples” a large spatial region and therefore responds sensitively to the transverse momentum of incident light.

Partially nonlocal structures are not without precedent. qBIC represents a well-known example, though the community has historically understood them primarily through the lens of finite Q factors, often overlooking the accompanying change in spatial coherence^11^. Physically, radiative leakage in a qBIC weakens the global spatial correlation of an ideal BIC, allowing individual structural units to regain partial independence. It is this very reduction in nonlocality that frees up spatial degrees of freedom and creates capacity for information encoding^12^. If this correlation were weakened to the extreme where adjacent units lose all coherence, the system would simply degenerate into a traditional local metasurface. However, it is important to note that the relationship between Q factor and nonlocality, while correlated, is not strictly equivalent. High-Q resonances can also arise in isolated single-unit structures with no inter-unit coupling (and hence no nonlocality)^13–15^. The nonlocality-Q correlation becomes operative specifically when coupling between units exists: in that regime, weaker radiation per unit implies larger lateral mode extent and stronger nonlocality.

Viewed through this unified framework, we can address our earlier question regarding the origin of design DoFs. They do not emerge from a perfect, cost-free superposition of two mechanisms, but rather reflect the recovery of spatial encoding capacity that was previously locked by unnecessarily strict periodicity. The additional spatial degrees of freedom become accessible once one recognizes that a practical qBIC does not require every unit cell to be identical. Instead, it requires only that the perturbations introduced remain compatible with the mode’s spatial coherence length. The achievement of this work lies in identifying a structural perturbation (the meta-notch) that maximally exploits this tolerance window: it is positioned at a field node of the BIC mode, and its variation is confined to a parameter range that preserves the topological encirclement mechanism for phase control.

Beyond its technical demonstration, this research reframes the design philosophy of micro- and nano-optics. It invites the community to move past the binary classification of pure metasurfaces versus pure photonic crystals, and instead to systematically explore the partially nonlocal regime. By quantifying the perturbation tolerance of nonlocal modes and deliberately designing within that tolerance, researchers can make informed trade-offs between Q factors and spatial degrees of freedom, or as this work shows, discover regimes where both coexist with minimal compromise. This perspective opens a new physical design space for advanced imaging, optical communication, analog computing, and the next generation of integrated optoelectronic devices^16–18^.
